# Investigating Power Density and the Degree of Nonlinearity in Intrinsic Components of Anesthesia EEG by the Hilbert-Huang Transform: An Example Using Ketamine and Alfentanil

**DOI:** 10.1371/journal.pone.0168108

**Published:** 2016-12-14

**Authors:** Feng-Fang Tsai, Shou-Zen Fan, Yi-Shiuan Lin, Norden E. Huang, Jia-Rong Yeh

**Affiliations:** 1 Department of Anesthesiology, National Taiwan University Hospital, Taipei, Taiwan; 2 Department of Anesthesiology, College of Medicine, National Taiwan University, Taipei, Taiwan; 3 Rey Institute for Nonlinear Dynamics in Medicine, Beth Israel Deaconess Medical Center, Harvard Medical School, Boston, MA, United States of America; 4 Center for Dynamical Biomarkers and Translational Medicine, National Central University, Taoyuan, Taiwan; 5 Research Center for Adaptive Data Analysis, National Central University, Taoyuan, Taiwan; CNRS, FRANCE

## Abstract

Empirical mode decomposition (EMD) is an adaptive filter bank for processing nonlinear and non-stationary signals, such as electroencephalographic (EEG) signals. EMD works well to decompose a time series into a set of intrinsic mode functions with specific frequency bands. An IMF therefore represents an intrinsic component on its correspondingly intrinsic frequency band. The word of ‘intrinsic’ means the frequency is totally adaptive to the nature of a signal. In this study, power density and nonlinearity are two critical parameters for characterizing the amplitude and frequency modulations in IMFs. In this study, a nonlinearity level is quantified using degree of waveform distortion (DWD), which represents the characteristic of waveform distortion as an assessment of the intra-wave modulation of an IMF. In the application of anesthesia EEG analysis, the assessments of power density and DWD for a set of IMFs represent dynamic responses in EEG caused by two different anesthesia agents, Ketamine and Alfentanil, on different frequency bands. Ketamine causes the increase of power density and the decrease of nonlinearity on γ-band neuronal oscillation, which cannot be found EEG responses of group B using Alfentanil. Both agents cause an increase of power density and a decrease of nonlinearity on β-band neuronal oscillation accompany with a loss of consciousness. Moreover, anesthesia agents cause the decreases of power density and nonlinearity (i.e. DWD) for the low-frequency IMFs.

## 1 Introduction

Electroencephalography (EEG) is noninvasive medical technique for monitoring and recording the electrical activity of brain. In clinical applications, Bi-spectral index (BIS) [1], audio evoked potential (AED) [2] and spectral entropy [3] perform ensemble assessments for quantifying the depth of anesthesia. However, the underlying pharmacological mechanisms of anesthesia agents are complicated. Different anesthesia agents cause different pharmacological reactions in a human brain, which also cause different dynamical patterns in surface EEG. Ensemble assessments are sufficient to provide a simple indicator representing the sedation status and level of consciousness, but they are insufficient to be a tool for characterizing the dynamic properties of EEG affected by different anesthesia agents. For the purpose to characterizing the dynamic properties of EEG, time-frequency analysis is commonly used to study the dynamics of an EEG signal in both time and frequency domains simultaneously [4, 5]. One of the most basic forms of time–frequency analysis is the short-time Fourier transform (STFT) [6], but more sophisticated techniques have been developed, notably wavelets [7]. In the traditional spectral analysis based on Fourier transform or wavelet, the spectra are derived from finite time periods, in which signals are assumed to be linear and stationary. However, EEG signals are totally nonlinear and non-stationary [8]. EEG signals contain complicated frequency modulations (FM) and amplitude modulations (AM) in the structure of data [9]. Both AM and FM are important dynamic features of nonlinear and non-stationary signals. But, the local AM and FM cannot be measured by traditional spectral analysis methods based on the assumptions of linearity and stationary. To overcome the drawbacks of traditional methods of time-frequency analysis, the innovative signal processing and analysis method named as Hilbert-Huang transform (HHT) [10] was used as the critical technique in this study. HHT works to decompose a nonlinear and non-stationary signal into a set of intrinsic mode functions (IMFs) by the method named as empirical mode decomposition (EMD), in which the instantaneous frequency and amplitude are derived by Hilbert transform as functions of time. The oscillations of instantaneous frequency and amplitude depict local and detailed AM and FM for an IMF. The function of EMD had been approved as an adaptive filter bank [11]. The spectra of instantaneous bandwidths and frequencies of IMFs are adaptive to the nature of data. It is convenient to map the instantaneous frequency bands into their correlated frequency bands (δ band: 0.5–3.5 Hz; θ band: 3.5-8Hz, α band: 8-13Hz; β band: 13–30 Hz; and γ band: 32-80Hz) according to the spectrum of EEG bandwidths and frequencies in clinical use and in research [12]. In the study for investigating the characteristics of white noise by EMD [13], a parameter of power density was defined to represent the power of an IMF. In this study, power density is used to represent the brain responses using the power of inter-wave amplitude modulation of an IMF on its corresponding bandwidth and frequency. In addition, the nonlinearity for an IMF is defined as a new assessment based on intra-wave frequency modulation [14]. In previous studies, the degree of nonlinearity had been used to assess the intra-wave frequency modulation as the characteristic parameter of waveform for nonlinear oscillations [15, 16]. The degree of nonlinearity reflects a new feature different from the intensity as the power of inter-wave amplitude modulation. Moreover, we also took a modification for the calculation of degree of nonlinearity to reduce the computing loading and complexity.

In additions, the evidence from anesthesia converges to suggest that loss of consciousness is associated with breakdown of cortical connectivity and thus of integration [17]. The functional connectivity of the frontoparietal association cortex is often reduced. The prime candidates for functional networks of the forebrain that play a critical role in maintaining the state of consciousness are those based on the posterior parietal-cingulate-precuneus region and the nonspecific thalamus [18]. Therefore, this investigation focused on the analysis of EEG signals at the frontal lobe (FP1). In the application of anesthesia EEG analysis, 56 patients with a low anesthetic risk requiring general anesthesia participated in this study. They were randomly divided into two groups. Ketamine was used for 28 patients of group A and Alfantnil was used for the other 28 patients of group B. Both assessments of power density and nonlinearity were used to assess the neuronal oscillations on different frequency bands during the first 10 minutes after the time point of anesthesia agent injection via the frontal EEG (channel FP1). We hypothesize that some changes of inter-wave amplitude modulations and intra-wave frequency modulations on specific intrinsic frequency bands are common responses correlated to the loss of consciousness. The word of ‘intrinsic’ means the frequency band determined adaptively to the nature of signals. Moreover, different anesthesia agents trigger their corresponding changes of EEG modulations on particular frequency bands. According to our findings, Ketamine triggers an increase of γ-band power (i.e. the power density of IMF1 from EEG) and a decrease of γ-band nonlinearity (i.e. the assessment of nonlinearity of IMF1). The γ-band responses of group B are totally different from those of group A. Moreover, there are some common responses on low-frequency bands of IMFs for groups A and B, which are considered to be the characteristics of EEG in anesthesia.

## 2 Materials

The present study was approved by the Institutional Review Board of National Taiwan University Hospital, and all participants provided written informed consent for EEG data analysis. There are no conflict issues in this study.

A total of 56 patients with a low anesthetic risk (American Society of Anesthesiologists Physical Status Classification I-II) requiring general anesthesia participated in this study. Prior to the surgical operation, the standard equipment for pulse oximetry, electrocardiography (EEG), BIS monitoring, and blood pressure monitoring were set up before anesthesia induction, following which recording was initiated. After monitoring patients for one minute, an anesthesiologist induced anesthesia using low-dose of Propofol. The frontal EEG signals were collected through a BIS module. Different anesthesia agents of Ketamine or Alfantnil were injected into the subjects, which were randomly sorted into two different groups, respectively, when an anesthesiologist confirmed that patients were consciousless. Surgical operations started at ten minutes after Ketamine/Alfentanil injection for recording the frontal EEG responses to different anesthesia agents.

## 3 Methods

In this section, we describe what is the function of a adaptive filter bank using the enhanced EMD method and the distribution of instantaneous frequency for each IMF. The correlated frequency bands of IMFs are determined according to the correlation between the distributions of instantaneous frequency and the spectra of bandwidths and frequencies in the clinical use.

### 3.1 Classical empirical mode decomposition (CEMD) & Hilbert-Huang transform (HHT)

The classical EMD (CEMD) proposed in 1998 by Huang et al. [10] is the first step of HHT for decomposing a nonlinear and non-stationary time series into a set of IMFs. The procedure to decompose a time series into IMFs is show as the followings:

1. Identify the local extrema (i.e. maxima and minima) of the time series *X(t)*
2. Connect local maxima/minima using cubic splines to generate an upper/lower envelop *e*_*max*_*(t)/e*_*min*_*(t)* of the time series.
3. Define the mean envelop as *m*_*1*_*(t) = (e*_*max*_*(t) + e*_*min*_*(t))/2*
4. The mean envelop is subtracted from the time series, providing the local oscillatory component *h*_*1*_*(t) = X(t)–m*_*1*_*(t)*.
5. Check whether the component satisfies the conditions [10] to be an IMF. If yes, the component is considered as first IMF denoted *C*_*1*_*(t) = h*_*1*_*(t)*. On the other hand, if *h*_*1*_*(t)* is not an IMF, consider the component as a new data to obtain a new component by repeating steps 1~5 until the new component satisfies the conditions to be an IMF.
6. Subtract the IMF from original time series to obtain the first residual *r*_*1*_*(t) = X(t)–C*_*1*_*(t)*. Consider the residual is a new time series and repeat steps 1~5 to obtain the next IMF until the residual is a monotonic time series.

After decomposition by EMD, the original time series is the sum of all IMFs and the final residual. Hilbert transform can be used as the second step of HHT to derive the analytical form $z_{i}(t)=C_{i}(t)+{j\;\tilde{C}}_{i}(t)=A_{i}(t)e^{j\theta_{i}(t)}$ for each IMF, where *A*_*i*_*(t)* is amplitude time series and *θ*_*i*_*(t)* the phase of the *i*th IMF. The differential of phase represents the instantaneous frequency.

### 3.2 The adaptive filter banks using the enhanced EMD method and the distributions of instantaneous frequency for the IMFs decomposed from EEG signal in sampling rate of 128 Hz

In this study, EEG signals were recorded in sampling rate of 128 Hz and decomposed into the first 6 IMFs by an enhanced EMD method. The mode-mixing problem is a major obstacle to the use of EMD on many signals [19, 20]. For the purpose to solve the mode-mixing problem, many enhanced methods, such as ensemble EMD (EEMD) [21] and complementary ensemble EMD (CEEMD) [22], had been suggested to filter out the intermittency, which causes the mode-mixing problem, by adding white noises into the original signals. As a different approach, masking signals were used to improve EMD for separate components that are similar in frequency that would be inseparable with CEMD techniques [23]. In this study, a set of four masking signals, sine waves with different predetermined initial phases, are used to solve the mode-mixing problem in the CEMD method and to avoid the residual of added white noise in the EEMD method.

In the enhanced EMD, the frequencies and amplitudes of masking signals are determined adaptively to the natures of signals. A pre-decomposition using the CEMD method for only IMF1 is used to determine the frequency and amplitude of masking signals used in the enhanced EMD method. In the enhanced method, a set of IMFs, *c*_*i*_*(t)*, decomposed from a signal, *x(t)*, can be derived by the following steps.

1. Derive the pre-decomposed IMF1 by the CEMD method.
2. Obtain the time series of instantaneous frequency and amplitude for the pre-decomposed IMF1 using Hilbert transform.
3. Determine the frequency of masking signals using the mean value of the distribution of instantaneous frequency, and determine the amplitude of masking signals using averaged amplitude of the pre-decomposed IMF1.
4. Generate four masking signals using the sine waves with amplitude and frequency determined by step 3. The initial phases of four masking signals are 0, π/2, π, and 3π/2.
5. Add masking signals into the original signal to generate four mixtures for formal decomposition by the CEMD, and decompose IMF1 from each mixture.
6. Obtain a new IMF *c*_*i*_*(t)* using the averaged mode function of four IMF1 decomposed from four mixtures.
7. Obtain the residual by removing the new IMF from the signal for decomposing next IMF until the residual is monotonic.

**Fig 1:**
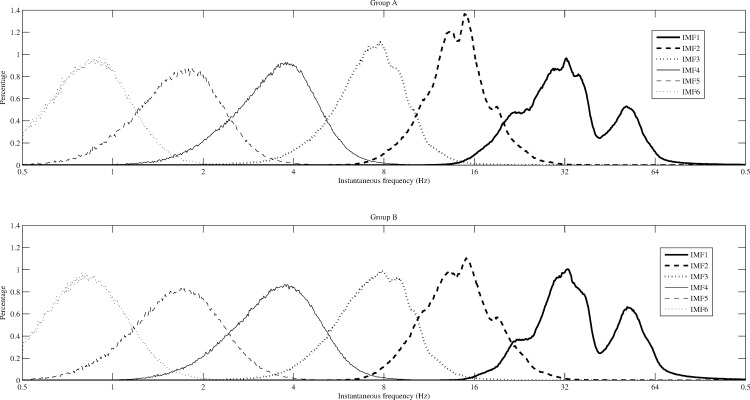
The distributions of instantaneous frequency for the first 6 IMFs decomposed from anesthesia EEG recordings of 56 subjects for two groups. Group A includes 28 subjects using Ketamine. Group B includes 28 subjects using Alfentanil.

The enhanced EMD method works well to solve the mode-mixing problem. In comparison with the performance of EEMD, low computing loading and no residual of added white noises are two advantages of the enhanced EMD in comparing with the functions of EEMD. In this study, anesthesia EEG signals were decomposed into the first 6 IMFs using the enhanced EMD method. The distributions of instantaneous frequencies for the six IMFs decomposed from EEG recordings from 56 subjects for two groups recruited in this study are shown in Fig 1.

According to the spectrum of EEG bandwidths and frequencies in clinical use and in research, IMF1 represents the γ band neuronal oscillation (> 30 Hz); IMF2 represents β band oscillation (13–30 Hz); IMF3 reflects α band oscillation (8-13Hz); IMF4 reflects θ band oscillation (3.5-8Hz); and IMFs 5–6 represents δ band oscillation (0.5–3.5Hz). The distributions of instantaneous frequency of the first 6 IMFs for two groups are similar.

### 3.3 Power density of a section of an IMF

The power density is a fundamental assessment of an intrinsic component (IMF). A short-term power density represents the magnitude of short-term fluctuation for an intrinsic component. In this study, a short-term power density for instant t is calculated using the following equation.

$$PD_{i}\left(t\right)=\frac{1}{p}∙\sum_{\tau =t-0.5p}^{\tau =t+0.5p}c_{i}\left(\tau \right)∙c_{i}\left(\tau \right)$$ (1)

where, *PD*_*i*_*(t)* is the power density at instant *t* of *i*-th IMF *c*_*i*_*(τ)*, and *p* is the segment length for the calculation of power density. Here, a segment length of 3 seconds was used for calculating power density.

### 3.4 The degree of nonlinearity based on intra-wave frequency modulation

In the HHT principle, the fluctuating of instantaneous frequency in one oscillation is regarded as intra-wave frequency modulation. The intra-wave frequency modulation is a unique definition of HHT [10, 14], in which frequency changes from time to time within an oscillation. The profile of an oscillation with intra-wave frequency modulation is not a pure sine or cosine wave. The intra-wave frequency modulation deforms the wave profile and the characteristic of the waveform deformation is not evalutable by traditional methods using Fourier spectral analysis and wavelet analysis.

According to Huang’s definition of nonlinearity [15], the assessment of intra-wave frequency modulation, which measures the deviation of the instantaneous frequency from the mean frequency in one oscillation based on the zero crossing period, is related to nonlinearity. To demonstrate the calculation of the assessment of intra-wave frequency modulation, a simple mathematic model using a generalized intra-wave frequency modulation as:

x(t)=cos(2πwt+δ∙sin(η∙2πwt)) (2)

where *x(t)* represents an frequency modulated signal with modulating signal *sin(η2πwt)*; *w* is the carrier frequency; *η* is the ratio of modulating to carrier frequency; and *δ* is a measure of strength of the frequency modulation.

**Fig 2:**
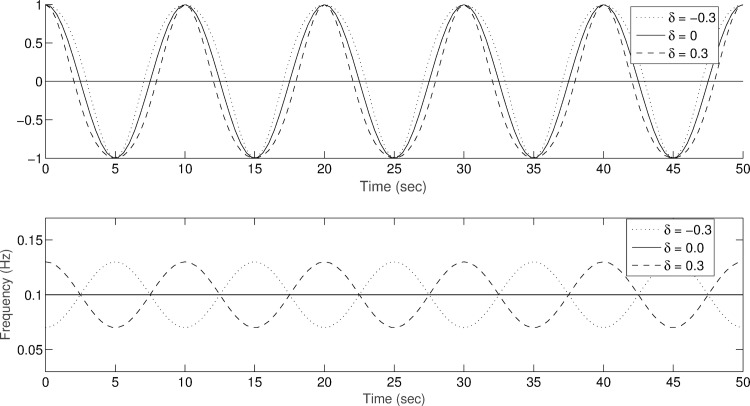
The simulated nonlinear wave and its frequency modulation for the simple mathematic nonlinear model using a generalized intra-wave frequency modulation with ratio of modulating to carrier frequency *η* = 1. (a) shows the simulated signals for strength of modulation *δ* = -0.3, 0, and 0.3; (b) shows the instantaneous frequency of the simulated signals.

Fig 2(A) shows the waveforms and instantaneous frequency of the simulated signals with intra-wave frequency modulation using the ratio of modulating to carrier frequency *η* = 1. The waveform distortion depends on the intra-wave frequency modulation. In the simulation, the instantaneous frequency of the modulated signal is 2π*w (1 + ηδ cos η2*π*wt)*. Therefore, the deviation of wave shape from the linear one (*δ* = 0), or the changes of instantaneous frequency (intra-wave modulations in a nonlinear system) from a constant value (for a linear system), can be used as a measure of nonlinearity. In 2013, Norden et al. suggested the deviation of intra-wave frequency modulation represents an assessment of degree of nonlinearity (DN), and it can be de calculated by the following equation [15]:

$$DN={\left[\frac{1}{n}\sum_{t=1}^{n}{\left(\frac{IF\left(t\right)-IF_{z}}{IF_{z}}\right)}^{2}\right]}^{1/2}$$ (3)

where, *DN* is the degree of nonlinearity; *IF(t)* is the instantaneous frequency for an IMF; n is the sample number of the segment; and *IF*_*z*_ is the mean frequency based on the zero crossing period.

**Fig 3:**
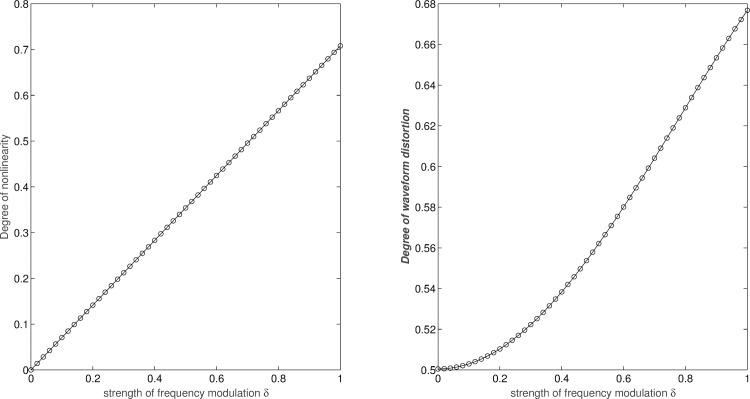
Degree of nonlinearity and DWD for the modulated signals of *η* = 1 and *δ* from 0 to 1.

In the numerical simulation, the strengths *δ* from 0 to 0.3 were used to modulate the intra-wave frequency of oscillations based on sine wave. Then, a linear relationship between the strength of frequency modulation and the degree of nonlinearity is shown as the Fig 3(A).

### 3.5 The degree of nonlinearity based on the profile of waveform distortion

Instantaneous frequency is necessary for understanding the detailed mechanisms frequency modulation of oscillation. Historically, instantaneous frequency was computed from analytic signal through the Hilbert transform. In facts, there are many difficulties for computing instantaneous frequency and many methods proposed to overcome the difficulties [24]. In this study, for the purpose to simplify the calculation of degree of nonlinearity based on the deviation of instantaneous frequency, the ratio of the power density to the power of envelope represents a characteristic feature of normalized oscillations, which contains the information of waveform distortion corresponding to its particular intra-wave frequency modulation. According to the results of our numerical simulations, the ratio of power density to the power of envelope is 0.5 for a linear wave (i.e. a sine wave). In this study, the ratio of power density to the power of envelope is named as degree of waveform distortion (DWD). The DWD values for the simple mathematic model of Eq (2) with ratio of modulating to carrier frequency *η* = 1 were calculated for the strength of frequency modulation *δ* from 0 to 1 as shown in Fig 3 The relationship between DN and DWD is positive and nonlinear.

In this study, DWD represents a different feature of waveform distortion from the power density, which represents the magnitude of oscillations during a time period in an IMF.

## 4 Results

### 4.1 Solve the mode-mixing problem by enhanced EMD using masking signals

**Fig 4:**
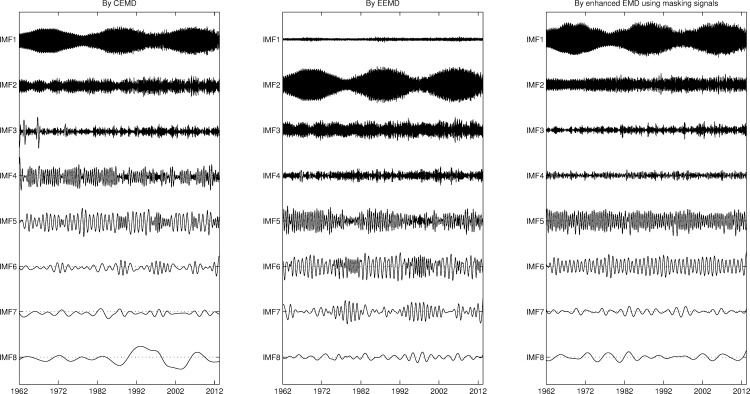
Three sets of IMFs decomposed from the daily length of day dataset by CEMD, EEMD, and the enhanced EMD using masking signals.

The mode-mixing problem is a serious issue for the applications using EMD. In this investigation, EEG signals were decomposed into sets of IMFs by the enhanced EMD algorithm using masking signals. To test the performance of the enhanced method, the daily length of day (LOD) dataset, which contains many different intrinsic oscillating cycles, was decomposed into sets of IMFs by the methods of CEMD, ensemble EMD and the enhanced EMD using masking signals as shown in Fig 4.

As shown in Fig 4, the mode-mixing phenomenon can be observed in IMF 4–6 for the set of IMFs decomposed by the CEMD; in IMF 6 & 7 for the set of IMFs decomposed by EEMD. The mode-mixing phenomenon is not found in those by the enhanced EMD using masking signals. IMF 1 decomposed by EEMD is a redundant mode function as a part of residual of added white noises. IMF6 in the set of IMFs decomposed by the enhanced EMD is an annual cycle, which is scattered into two different IMFs for both set of IMFs decomposed by the CEMD and EEMD. These results prove that the enhanced EMD using masking signals works promisingly to solve the mode-mixing problem.

### 4.2 Frequency-depend power density for different groups

**Fig 5:**
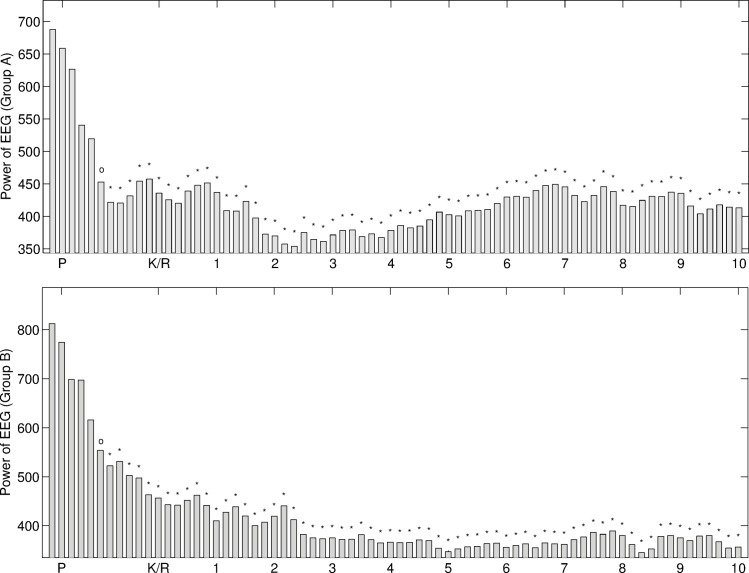
The power of the reconstructed EEG signals using the first 6 IMFs for two groups using Ketamine/Alfentanil. The time point before the mark of ‘P’ is the baseline; the mark of ‘P’ is the start point of propofol infusion; and the mark of ‘K/R’ is the time point of anesthesia agent injection. The marks of ‘o’ represent the p-value of statistical test in comparison with baseline lower than 0.05, and the marks of ‘*’ represent the p-value lower than 0.01.

In the study of anesthesia EEG analysis, a sliding window with a size of 10 seconds was used to quantify the power density and DWD for each IMF. One-minute EEG signal before anesthesia induction is selected to be the baseline as pre-anesthesia EEG. For the purpose to observe the process of losing consciousness, ten check points were set up according to the individual time period from anesthesia induction to lost consciousness. The first ten minutes of EEG recording after Ketamine/Alfentanil injection were analyzed using the enhanced EMD method. Since the instantaneous frequency distribution of IMF 7 is lower than 0.5 Hz, only the first 6 IMFs were used to reconstruct the EEG signals. The oscillations within frequency band lower than 0.5 Hz and the long-term trend in EEG signals were filtered out in this study. Fig 5 shows the power of the reconstructed EEG for baseline, during the period of losing consciousness, and ten-minutes after anesthesia agent (Ketamine for group A and Alfentanil for group B) injection. EEG power for both groups became significantly different from the baseline at the forth check point after anesthesia induction. For group A, EEG power keeps low during the first two minutes and then increases at the third minute after Ketamine injection. For group B, EEG power keeps low during the anesthesia stage. EEG power during the anesthesia stage is significantly lower than that of baseline for both groups.

**Fig 6:**
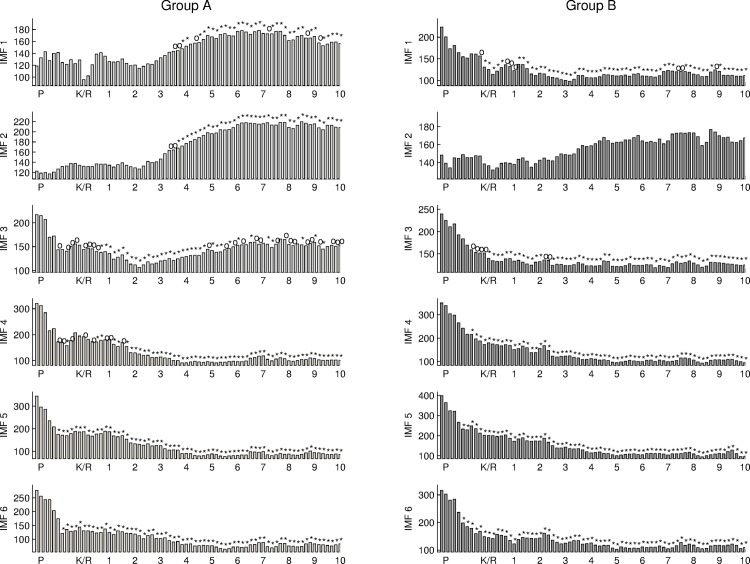
The power density of the first 6 IMFs for two groups. The time point before the mark of ‘P’ is the baseline; the mark of ‘P’ is the start point of propofol infusion; and the mark of ‘K/R’ is the time point of anesthesia agent injection.

For the purpose to detail the changes in EEG signals, the power densities for the first 6 IMFs from EEG are shown in Fig 6. For both groups, the power densities of IMF 4–6 during anesthesia stage are significantly lower than those of baseline. The power density of IMF1 during anesthesia stage is significantly lower than that of baseline for group A, but the power density of IMF1 significantly increases after the third minutes after Ketamine injection for group B. The changes in power densities of the first 3 IMFs for two groups are total different.

**Fig 7:**
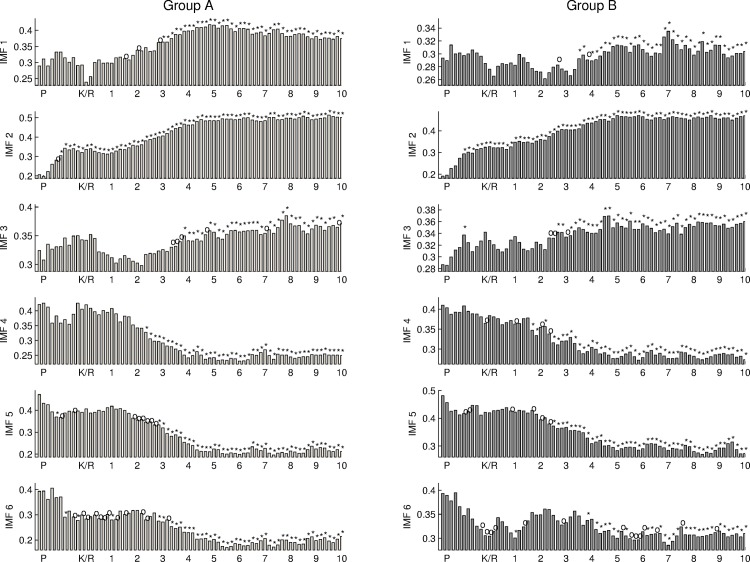
The normalized power density of the first 6 IMFs for two groups.

In addition, the power densities of the first 6 IMFs are normalized using the EEG power to represent the power distribution on different frequency bands as shown in Fig 7. The normalized power density of IMF2 after the forth check point after anesthesia induction significantly different from that of baseline. This implies that the normalized power density of IMF2 is a potential assessment correlated to the level of consciousness. During the anesthesia stage, the normalized power densities of IMF 4–6 are significantly lower than that of baseline for both groups.

### 4.3 Results using degree of waveform distortion

**Fig 8:**
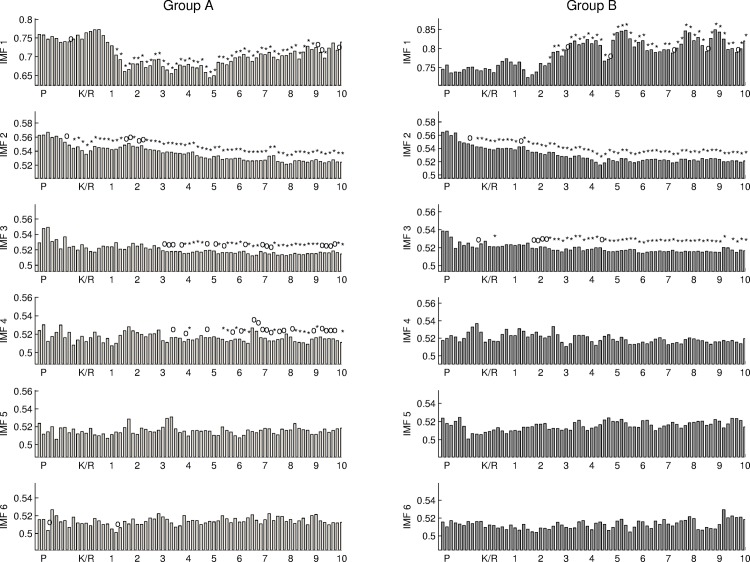
The DWD values of the first 6 IMFs for two groups.

In this section, DWD was used to quantify the nonlinearity of the IMFs. As shown in Fig 8, DWD values of IMF2 for both groups significantly decrease with time. During the time period from anesthesia induction to the surgical operation, DWD of IMF 2 and 3 gradually decrease until steady state. This result implies that the level of consciousness is correlated to the power density and degree of nonlinearity in IMF 2 and 3. A high power density accompany with low nonlinearity of IMF2 reflects a low level of consciousness. However, the DWD of IMF1 for group A is significantly different from that for group B when different anesthesia agents were injected into the human bodies. Ketamine causes an increase of power density and a decrease of nonlinearity for IMF1, but Alfentanil causes a decrease of power density (as shown in Fig 6) and an increase of nonlinearity for IMF1. These results imply that two anesthesia agents cause different responses in EEG signals, which reflect different underlying physiological mechanisms in a human brain.

## 5 Discussions and Conclusions

In this study, an enhanced EMD method using masking signals is proposed and used in anesthesia EEG analysis. Both frequencies and amplitude of masking signals are determined according the nature of EEG signals. A pre-decomposition was used to determine the frequency and amplitude of conjugate masking signals used for decomposing an IMF formally. This enhanced EMD works out high performance for solving the mode-mixing problem. Then, the enhanced EMD method worked as a good dyadic filter bank for decomposing EEG signals into sets of IMFs. Moreover, the enhanced EMD method also keeps the nature of nonlinearity in the decomposed intrinsic components (i.e. IMFs).

In many previous study of amplitude-frequency studies based on empirical mode decomposition, a scaling behavior can be quantified by marginal spectral analysis in turbulences [25] and fractal Gaussian noises [11]. Multiscale high-order moments introduced by Huang et al. [25] are candidates for assessing local scaling property of EEG signals with a limitation of short data length. Moreover, we checked the distributions of instantaneous frequency for the first six IMFs using intermittency measure introduced by Farge [26]. The moment values of skewness and kurtosis are used to assess the intermittencies for three sets of IMFs decomposed by CEMD, EEMD, and enhanced EMD respectively. The mean values of skewness for the first 5 IMFs decomposed by CEMD are 0.10, 0.16, -0.01, 0.11 & 0.40; the values derived by EEMD are 2.10, 1.75, 0.59, 0.12 & 0.18; the values derived by the enhanced EMD are 1.39, 0.87, 0.11, -0.07 & -0.08. The mean values of kurtosis for the first 5 IMFs decomposed by CEMD are 2.60, 3.64, 3.79, 4.06 & 5.88; the values derived by EEMD are 17.22, 21.97, 11.81, 5.96 & 5.17; the values derived by the enhanced EMD are 11.48, 11.72, 7.04, 4.58 & 4.32. These results show that the instantaneous frequency distribution for each IMF decomposed by CEMD is similar to a normal distribution. Those for first 4 IMFs decomposed by the enhanced EMD are centralized distributions. The overlap between two sequential IMFs decomposed by CEMD is larger than that by the enhanced EMD. Centralized instantaneous frequency distributions and small overlaps represent the mod-mixing problem is diminished by the enhanced method.

In this study, two different anesthesia agents were used in the general anesthesia. Power density and DWD are used to quantify the strength and nonlinearity of IMFs with different frequency bands. According to the analysis results using power and power density, the common changes from conscious to consciousless for two groups can be clearly observed via the normalized power density of IMF2 and IMF 4–6. In general, EEG power in anesthesia is lower than that of baseline (i.e. awareness). The power density of IMF1 reflects significant difference between the dynamical characteristics of EEG signal for two groups. Ketamine causes a significant increase of power density in IMF1 and Alfentanil does not.

On the other hand, nonlinearity represents a new feature different from power density. Anesthesia causes significant decreases of nonlinearity in comparison with baseline on IMF 2–3. Ketamine causes a decrease of nonlinearity and an increase of power density in IMF1 before the start of surgical operation. The nonlinearity of IMF1 became unstable because of surgical actions. In this study, what causes the nonlinearity and power density of IMF1 during the time period of taking surgical operation is not clear. It is hard to evaluate the effects caused by surgical stresses and the medicine. In this study, we found some common responses in frontal EEG during a general anesthesia and some particular responses correlated to the pharmacological effects of Ketamine.

In conclusions, the EMD method is an innovative algorithm for analyzing the nonlinear and non-stationary real-world signals, such as EEG signals. However, the mode-mixing problem is always a big issue for the applications using EMD. In this study, we suggest the conjugate masking signals with frequency and amplitude adaptive to the nature of the decomposed component can be used to overcome the mode-mixing problem as a big advantage of this study. Moreover, the use of an assessment of nonlinearity is another advantage of this study.
